# Rationale, design, and protocol for a hybrid type 1 effectiveness-implementation trial of a proactive smoking cessation electronic visit for scalable delivery via primary care: the E-STOP trial

**DOI:** 10.1186/s12875-023-02205-3

**Published:** 2023-11-29

**Authors:** Margaret C. Fahey, Amy E. Wahlquist, Vanessa A. Diaz, Marty S. Player, Noelle Natale, Katherine R. Sterba, Brian K. Chen, Eric D. A. Hermes, Mathew J. Carpenter, Jennifer Dahne

**Affiliations:** 1 Department of Psychiatry & Behavioral Sciences, Medical University of South Carolina, MSC 955, 86 Jonathan Lucas Street, Charleston, SC 29425 USA; 2 Center for Rural Health Research, East Tennessee State University, Johnson City, TN USA; 3 Department of Family Medicine, Medical University of South Carolina, Charleston, SC USA; 4 Department of Public Health Sciences, Medical University of South Carolina, Charleston, SC USA; 5 Arnold School of Public Health, University of South Carolina, Columbia, SC USA; 6 Department of Psychiatry, Yale University School of Medicine, New Haven, CT USA; 7 Hollings Cancer Center, Medical University of South Carolina, MSC 955, 86 Jonathan Lucas Street, Charleston, SC 29425 USA

**Keywords:** Smoking cessation, Electronic visit (e-visit), Electronic health records, Primary care

## Abstract

**Background:**

Cigarette smoking remains the leading cause of preventable disease and death in the United States. Primary care offers an ideal setting to reach adults who smoke cigarettes and improve uptake of evidence-based cessation treatment. Although U.S. Preventive Services Task Force Guidelines recommend the 5As model (Ask, Advise, Assess, Assist, Arrange) in primary care, there are many barriers to its implementation. Automated, comprehensive, and proactive tools are needed to overcome barriers. Our team developed and preliminarily evaluated a proactive electronic visit (e-visit) delivered via the Electronic Health Record patient portal to facilitate evidence-based smoking cessation treatment uptake in primary care, with promising initial feasibility and efficacy. This paper describes the rationale, design, and protocol for an ongoing Hybrid Type I effectiveness-implementation trial that will simultaneously assess effectiveness of the e-visit intervention for smoking cessation as well as implementation potential across diverse primary care settings.

**Methods:**

The primary aim of this remote five-year study is to examine the effectiveness of the e-visit intervention vs. treatment as usual (TAU) for smoking cessation via a clinic-randomized clinical trial. Adults who smoke cigarettes are recruited across 18 primary care clinics. Clinics are stratified based on their number of primary care providers and randomized 2:1 to either e-visit or TAU. An initial baseline e-visit gathers information about patient smoking history and motivation to quit, and a clinical decision support algorithm determines the best evidence-based cessation treatment to prescribe. E-visit recommendations are evaluated by a patient’s own provider, and a one-month follow-up e-visit assesses cessation progress. Main outcomes include: (1) cessation treatment utilization (medication, psychosocial cessation counseling), (2) reduction in cigarettes per day, and (3) biochemically verified 7-day point prevalence abstinence (PPA) at six-months. We hypothesize that patients randomized to the e-visit condition will have better cessation outcomes (vs. TAU). A secondary aim evaluates e-visit implementation potential at patient, provider, and organizational levels using a mixed-methods approach. Implementation outcomes include acceptability, adoption, fidelity, implementation cost, penetration, and sustainability.

**Discussion:**

This asynchronous, proactive e-visit intervention could provide substantial benefits for patients, providers, and primary care practices and has potential to widely improve reach of evidence-based cessation treatment.

**Trial registration:**

NCT05493254.

**Supplementary Information:**

The online version contains supplementary material available at 10.1186/s12875-023-02205-3.

## Introduction

Cigarette smoking remains the leading cause of preventable disease and death in the United States (U.S.), resulting in 480,000 premature deaths each year [1, 2]. Despite nationwide progress in reducing smoking prevalence, 28.3 million U.S. adults continue to smoke cigarettes [3]. Smoking cessation significantly reduces the risk of premature death, as well as risk for a number of adverse health effects (e.g., cardiovascular diseases, chronic obstructive pulmonary disease, cancer) [1]. Although two-thirds of adults who smoke cigarettes want to quit, fewer than one-third use an evidence-based approach to support their quit attempts [1, 4]. As a result, fewer than one in ten adults who smoke cigarettes successfully quit each year [4]. Integrating cessation services into existing healthcare infrastructure can be a scalable, cost-effective, and efficacious way to widely reach adults who smoke cigarettes and improve the uptake of evidence-based cessation treatments [1].

Primary care offers an ideal healthcare setting in which to deliver cessation services given that the vast majority (> 70%) of adults who smoke cigarettes visit a primary care provider (PCP) annually [5–7]. Although the U.S. Preventive Services Task Force Guidelines recommend the use of the 5As model (Ask, Advise, Assess, Assist, Arrange) for cessation treatment in primary care [5], there are many barriers to its implementation (e.g., lack of provider time, confidence, and familiarity with guidelines) [8–10]. A U.S. nationwide lung cancer screening trial assessed the provision of cessation treatment among participants in primary care, finding that delivery of the 5As became less common as steps progressed: Ask smoking status (77.2%), Advise quitting (75.6%), Assess motivation (64.4%), Assist with referrals (56.4%), and Arrange follow-up (10.4%) [11]. Proactive, automated tools that can address barriers to delivery of evidence-based smoking cessation treatment within primary care are clearly needed.

To abide by the Centers for Medicare and Medicaid Services’ (CMS) Meaningful Use Guidelines [12, 13], primary care practices are required to maintain electronic health records (EHRs) with smoking status data for all patients. Therefore, EHRs offer a means via which to proactively identify patients who are smoking cigarettes and facilitate connection to high-quality, evidence-based treatment from a patient’s own PCP. Existing proactive automated cessation approaches within primary care have more commonly outsourced treatment to external sources (e.g., automated voice recognition messages, tobacco quit lines, text messaging) [14–16]. However, delivering treatment from one’s own PCP (rather than outsourcing treatment) affords the unique ability to capitalize upon the trust between adults who smoke and their PCPs [17], which is predictive of greater adherence to care [18]. Though few prior studies have evaluated automated delivery of cessation treatment from a patient’s own PCP, studies that have (including from our own group) have shown clinical benefit. For example, in one prior trial, asynchronous cessation care provided by one’s PCP via patient portals doubled quit rates compared to portal messaging delivering standard advice to quit [19]. Thus, cessation treatment approaches delivered in an automated fashion via the EHR from a patient’s own PCP could help to address barriers to receipt of cessation treatment and improve cessation outcomes for primary care patients.

An electronic visit (e-visit), which can be delivered automatically via the EHR, could be a fitting telehealth treatment to universally and proactively deliver cessation treatment to all adults who smoke cigarettes via primary care [20]. E-visits are embedded in the most common EHRs and offer a secure platform through which patients can remotely supply providers with health information. Providers in turn can deliver personalized treatment. Recent studies have concluded that implementation of e-visits within primary care could divert 20% of in-person visits to electronic communication [21] and eliminate PCP shortages nationwide [22]. Asynchronous e-visits enable PCPs to respond at a time that is suitable for them, eliminating in-session time constraints. Thus, PCPs can deliver tailored treatment to large numbers of patients [23, 24]. E-visit invitations can be automated and sent in bulk via the EHR to all patients who meet treatment criteria. After accepting an e-visit invitation, the patient completes a questionnaire that may include built-in algorithms to facilitate treatment decision-making. All e-visit outcomes are sent to the PCP (or another medical team member) via the EHR. Upon reviewing the e-visit, the PCP will recommend a treatment plan. If medications are indicated, the PCP can e-prescribe the medication to the patient’s pharmacy. Prior studies of asynchronous e-visits (for other health conditions) via primary care have found high satisfaction among both patients and PCPs and that such e-visits can be delivered efficiently (average time for patient completion = 8.3 min, average time for PCP to review and respond = 3.6 min) [25].

Our team previously developed and preliminarily evaluated an asynchronous smoking cessation e-visit for delivery via primary care and demonstrated via a pilot trial that this intervention may be a feasible and efficacious approach to extend the reach of cessation treatment [26]. During the smoking cessation e-visit, an initial baseline evaluation gathers information asynchronously about a patient’s smoking history and motivation to quit, and an evidence-based clinical decision support algorithm determines the best FDA-approved cessation medication (i.e., nicotine replacement therapy [NRT], varenicline, bupropion) to prescribe. A one-month follow-up e-visit clinically assesses cessation progress consistent with the fifth “A” to provide follow-up. Pilot trial outcomes indicate that at three-month follow-up, patients who received the e-visit intervention were 4.7 times more likely to have used an FDA-approved medication, 4.1 times more likely to have reduced their cigarettes per day by > 50%, and 4.2 times more likely to report 7-day point prevalence abstinence (PPA) [26].

This paper describes the rationale, design, and protocol for an ongoing five-year Hybrid Type I effectiveness-implementation trial that will simultaneously assess effectiveness of the e-visit intervention for smoking cessation as well as implementation potential across 18 primary care clinics within South Carolina. The e-visit intervention evaluated in this trial aims to improve cessation treatment access for adults who smoke cigarettes, allow PCPs to more efficiently deliver reimbursable smoking cessation services, and improve health care systems’ delivery of cessation treatment.

## Methods

### Study aims

The primary aim of this trial is to examine the effectiveness of an e-visit intervention vs. treatment as usual (TAU) within primary care for smoking cessation across 18 practices in South Carolina via a clinic-randomized clinical trial. Primary outcomes include 1) evidence-based smoking cessation treatment utilization (medication, psychosocial cessation counseling), 2) reduction in cigarettes per day, and 3) biochemically verified 7-day point prevalence abstinence (PPA) at six-month follow-up. We hypothesize that patients randomized to the e-visit condition will have significantly better cessation outcomes relative to TAU. The secondary aim is to evaluate e-visit implementation outcomes at patient, provider, and organizational levels. Implementation outcomes will be assessed using a mixed-methods approach and will follow Proctor’s Framework [27, 28] by evaluating acceptability, adoption, fidelity, implementation cost, penetration, and sustainability.

### Aim 1 effectiveness trial design

**Fig. 1:**
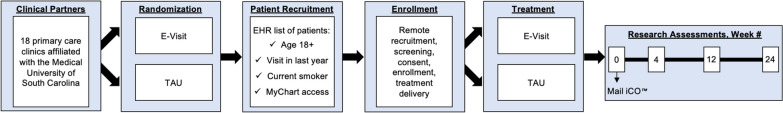
Aim 1 effectiveness randomized controlled trial study design

At trial outset, clinics are stratified based on their number of PCPs (1–4 vs > 4) and randomized 2:1 via a HIPAA compliant electronic database (REDCap) [29, 30] hosted by the South Carolina Clinical and Translational Science (SCTR) Institute at MUSC [30, 31] to receive either e-visit or TAU (see Fig. 1). Following enrollment, participants complete baseline assessments and receive their clinic-randomized intervention. A participant’s clinic is based on the location of their last primary care visit. Participants complete follow-up assessments at one, three, and six-months post-enrollment.

### Study setting & recruitment

Participants are recruited from primary care clinics affiliated with the Medical University of South Carolina (MUSC). Recruitment sites include 18 unique clinics which collectively treat more than 76,000 patients annually. These clinics are distributed across the South Carolina counties of Charleston, Dorchester, and Berkeley and all serve Health Professional Shortage Area (HPSA)-designated low-income populations [32]. See Table 1 for further detailed clinic-level information.

**Table 1:** Description of Medical University of South Carolina (MUSC) primary care clinics

Primary Care Clinic	# of Providers	HPSA Score	Rurality Status	Size
Clinic #1	1	9	Partially Rural	Small
Clinic #2	1	9	Partially Rural	Small
Clinic #3	2	9	Partially Rural	Small
Clinic #4	2	9	Partially Rural	Small
Clinic #5	3	14	Partially Rural	Small
Clinic #6	3	9	Partially Rural	Small
Clinic #7	3	9	Partially Rural	Small
Clinic #8	3	9	Partially Rural	Small
Clinic #9	4	12	Non-rural	Small
Clinic #10	5	9	Partially Rural	Large
Clinic #11	5	9	Partially Rural	Large
Clinic #12	6	16	Non-rural	Large
Clinic #13	6	14	Partially Rural	Large
Clinic #14	6	14	Partially Rural	Large
Clinic #15	7	9	Partially Rural	Large
Clinic #16	7	9	Partially Rural	Large
Clinic #17	12	9	Partially rural	Large
Clinic #18	15	9	Partially rural	Large

^*^All clinics serve Health Professional Shortage Areas (HPSA)-designated low-income populations. HPSA score was developed by the National Health Service Corps in determining priorities for the assignment of clinicians. Scores range from 0 to 26, with a higher score indicating greater priority for clinicians. Large clinic = clinic with more than 4 providers of any type

Participant recruitment occurs proactively and remotely. Leveraging EHR data, potential participants are identified via a study recruitment report generated by MUSC’s Biomedical Informatics Center (BMIC) (consistent with Institutional Review Board [IRB] procedures). MUSC patients ≥ 18 years of age, identified as smoking cigarettes, and who have seen a PCP within the past two months, or with an upcoming appointment in the next month, at one of the 18 eligible primary care clinics are included on the recruitment report. The following methods are used to cold-contact participants: MyChart (i.e., MUSC’s EHR patient portal), e-mail, text messaging, and phone call. The total target sample size is 672 participants.

### Participant screening, eligibility and enrollment

#### Screening

Identified patients are sent proactive study invitations that provide information about the study and a link to complete an online screening via REDCap. Invitations are first sent via MyChart message. If the patient does not respond to the MyChart screener within 72 h, they are sent an invitation via email, followed by text and then phone call. A total of three follow-up invitations are sent following the initial MyChart message. Follow-up invitations are sent once per day on consecutive days starting 72 h after the MyChart message is sent. If a participant completes the screener or informs study staff they are not interested in the study, invitations are ceased. Between 50–60 initial study invitations are sent each week.

#### Eligibility

Inclusion criteria include: 1) currently smoking defined as 5 + cigarettes/day for 20 or more days in the past 30 for the last 6 + months, 2) aged 18 years or older, 3) enrolled in MyChart or willing to enroll 4) possesses a valid e-mail address that is checked daily to access assessments and MyChart messages, 5) owner of an iOS or Android smartphone to provide remote carbon monoxide (CO) biochemical verification at follow-up assessments, 6) has a valid mailing address, and 7) English fluency. Exclusion criteria includes use of an FDA-approved cessation medication in the past 7 days.

#### Informed consent

Remote electronic informed consent (e-consent) is obtained via REDCap [33]. Participants receive a link to the REDCap e-consent form that they can review and sign, paired with a phone or video call with IRB-approved research staff to answer questions. As smartphone ownership is a study inclusion criterion, all eligible participants have internet access and thus access to the e-consent form.

### Smoking cessation electronic visit (E-Visit) intervention

Following screening and consent, participants recruited from clinics assigned to the e-visit condition are automatically linked to initiate the asynchronous smoking cessation e-visit via MyChart. All costs of the e-visit (i.e., $25 charge per e-visit paid to the clinic) are covered by the study during the effectiveness trial. The baseline e-visit gathers information about cigarette smoking history and motivation to quit, and an algorithm determines the best FDA-approved cessation medication (i.e., nicotine replacement therapy [NRT], varenicline, bupropion) to recommend to the patient. This algorithm is based on our team’s prior research [34–36] and evidence-based guidelines [5]. Branching logic is used to prioritize the most efficacious medications (i.e., varenicline and combination NRT), while tailoring recommendations based on contraindications and patient preference. The outcome is a medication recommendation displayed to the patient with a personalized rationale as to why the medication was selected. All medication recommendations are provided in conjunction with a referral to the tobacco quit line for psychosocial counseling and a digital copy of National Cancer Institute’s *Clearing the Air: Quit Smoking Today* [37]. Patients can agree with the recommendation or request a different treatment. E-visit results are automatically sent to the patient’s PCP’s in-basket where all information provided by the patient, including the algorithm-recommended treatment and the patient’s preferred treatment (if applicable), are displayed. Providers then 1) open the e-visit from their in-basket, 2) review the e-visit and its algorithm outcome (e.g., medication recommendation or patient requested medication if applicable), 3) review patient EHR chart for contraindications for that outcome, 4) agree or disagree with the treatment, 5) respond to the patient via MyChart with instructions, and 6) e-prescribe medication (if indicated). A patient’s own PCP has seven calendar days to respond. If the e-visit is not responded to within this time frame, it is routed to a central pool of MUSC providers who are trained in review of and response to completed e-visits.

All e-visit participants are scheduled for an asynchronous follow-up e-visit one month later to assess progress toward cessation and troubleshoot barriers (consistent with the 5As guidelines to Arrange follow-up). The one-month follow-up e-visit assesses current self-reported smoking status, quit attempts in the last month, and quit duration. Subsequently, the participant reports: 1) whether they received a cessation medication following baseline, 2) whether they are currently taking the medication, and 3) whether they have any questions/concerns. Participants are asked if they are interested in any other treatment options, including a medication refill. Those requesting a different treatment option are queried with the same series of questions from the baseline e-visit to determine contraindications. Results are sent to providers for review and response in the same manner as the baseline e-visit.

#### Medication considerations

Varenicline, a class C medication, may be prescribed as a result of the e-visit. Because risks during pregnancy related to varenicline are unknown, all participants who report female sex at screening receive additional questions to assess for current pregnancy and childbearing potential. Female patients are asked if they are pregnant or planning to become pregnant in the next six months. Those reporting no current or planned pregnancy, as well as no history of hysterectomy or surgical sterilization, are then asked the following 1) “Are you currently lactating?” (Yes/No) 2) “In the last 12 months, have you had a menstrual period?” (Yes/No), and 3) “Are you currently using a form of birth control that would cause you to not have had a menstrual period within the last year?” (Yes/No). If patients respond yes to any of these items, they are asked if they would be willing to complete a pregnancy test that will be mailed to them. These patients then receive a REDCap form via email and have three days to verify (with signature) that they completed the test and to confirm their pregnancy test results. If the participant reports either current or planned pregnancy, or reports a positive pregnancy test, they will not receive varenicline within the e-visit algorithm.

All medications are prescribed on label to the patient’s pharmacy on record and billed as in usual practice (i.e., to the patient’s insurance if insured), consistent with procedures from our pilot trial [26]. Participants are not required to obtain their prescribed medication from their pharmacy or to take the medication as part of study participation. Whether a participant receives their prescribed medication and reasons for non-receipt (e.g., medication cost) are tracked as a study outcome via self-report at follow-up assessments.

#### Provider training

Throughout the first three months following study startup, the study team (VD, MP) provided training on the smoking cessation e-visit to all PCPs affiliated with e-visit assigned clinics during departmental meetings. Because PCPs at MUSC already respond to e-visits as part of their clinical practice, study training focuses on the specific use of the smoking cessation e-visit and its decision-support algorithm. Trainings are recorded and distributed following meetings. A brief video describing the workflow and tip sheets with overviews of the e-visit functionality are also sent to providers. Tip sheets include information on smart phrases developed to improve the ease with which e-visits can be responded to (e.g., with information regarding varenicline dosing and links to additional information). These procedures are consistent with current training approaches to implement new clinical workflows in ambulatory practice. Training recordings and tip sheets are provided to new hires in e-visit clinics upon onboarding. Once a PCP receives their first e-visit to complete, the video and tip sheet are sent to them again via EHR. PCPs are also offered the opportunity to meet with study team (VD, MP) to review the e-visit protocol if they have any further questions.

### Treatment as usual (TAU)

TAU mimics existing usual care within primary care for smoking cessation treatment. Participants recruited from clinics assigned to TAU will be sent via MyChart a link to a screen that includes information on the state tobacco quit line, education about the importance of quitting, and a recommendation to contact their PCP to discuss quitting smoking. This same approach was also utilized in our pilot work [26].

### Assessments

Assessments occur at baseline as well as at one, three, and six-months post enrollment for participants in both treatment conditions. For all follow-up assessments, participants are text messaged and/or emailed (based on their preference) a REDCap link, accessible via smartphone. Assessments are estimated at 20 min each. Participants are compensated $20 in electronic gift codes for completion of each follow-up assessment, $20 for submission of CO at each follow-up, and a $100 bonus for completing all follow-up assessments (including self-report questionnaires and CO). A subgroup of participants in the e-visit condition will be invited to earn an additional $20 by participating in post-study interviews (to evaluate implementation determinants and outcomes).

#### Patient and cigarette smoking characteristics

At baseline, all participants report sociodemographic characteristics and complete measures assessing digital literacy (Mobile Device Proficiency Questionnaire (MDP-Q) [38], eHealth Literacy Scale [39], Computer Proficiency Questionnaire [40]) and depressive symptoms [Patient Health Questionnaire-8 [41]]. Cigarette smoking, use of other tobacco products (e.g., electronic cigarettes), and quit attempts/quit duration are assessed at baseline and at each follow-up using a 7-day timeline follow back [42, 43]. Nicotine dependence is assessed via the Fagerstrom Test of Nicotine Dependence [44], and participants also report motivation to quit and confidence in quitting using a modified Contemplation Ladder (range 0–10) [45] at baseline. Self-reported smoking is biochemically verified via breath CO, with abstinence defined as CO of ≤ 4 ppm at all follow-up assessments [46]. Self-report and CO data will be utilized together to determine 7-day PPA.

#### Treatment utilization

At each follow-up, participants in both conditions are queried for: 1) use of cessation treatment (medication and/or psychosocial counseling) since last assessment, 2) how medication was obtained, and 3) receipt of the 5As from their PCP [47].

#### Confounders of carbon monoxide (CO)

At all assessments, participants report combustible cannabis use, secondhand smoke exposure, and environmental CO exposure within the last 24 h to account for factors that may falsely inflate CO.

#### Remote biochemical verification

Because the e-visit is delivered remotely and the trial is conducted remotely, biochemical verification of smoking must also be completed remotely for all participants. Following enrollment, participants are mailed an iCOquit™ Smokerlyzer (personal breath CO monitor) [48]. All participants receive their iCOquit™ device via mail prior to their one-month follow-up in anticipation of having CO assessments at all follow-up timepoints. Along with their device, participants are also mailed a handout with information about how to use their iCOquit™ device. Participants are asked to download the iCOquit™ app and register their iCOquit™ device through the app prior to follow-ups. At each follow-up (one, three, six-months), participants complete a breath sample and use the share feature in the app which sends results to study team via email. Participants can also take a screenshot of their results and send to study team via text message or e-mail directly. All iCOquit™ data is entered and stored in REDCap by study team.

### Sample size justification

Our primary effectiveness outcome is cessation, defined as 7-day CO-verified PPA at six-months. Preliminary data from our pilot trial [26] demonstrated 7-day self-reported PPA rates at three-months of 21.7% and 6.3% for e-visit and TAU groups, respectively. Although we expect similar group differences in abstinence, we expect that 7-day PPA rates will be somewhat lower at six-months for both groups and conservatively estimate these rates to be 18% (e-visit) and 4% (TAU). In addition, we expect some degree of intra-clinic and intra-provider correlation (i.e., intraclass correlation [ICC]), where patients who “see” the same provider at the same clinic are correlated. We estimate this at 0.013 for providers, based on previous site-randomized primary care studies [49, 50]. We expect that not every MUSC PCP will have a patient enrolled in the trial and estimate that 67% of PCPs (103 PCPs) will have enrolled patients. Based on our pilot trial, we plan for attrition of 25% [26]. Using an ICC of 0.013 and an a priori significance (alpha) level of 0.05, (assuming 103 PCPs will have participating patients and inflating by 25% for attrition), a total sample of 672 (448 e-visit, 224 TAU) would have sufficient power (> 80%) to detect differences of 18% vs. 4% respectively, in 7-day PPA at six months.

Other trial outcomes include cessation treatment utilization and reduction in cigarettes per day by at least 50%. In our pilot trial, treatment utilization rates at three-months were higher in the e-visit group compared to TAU (60.9% vs. 25%, respectively); similarly, reduction in cigarettes per day was higher in the e-visit group (65.2% vs. 31.3%, respectively) [26]. With sample sizes of *n* = 448 in the e-visit group and *n* = 224 in the TAU group, we will have more than sufficient power to see similar differences, and in fact, smaller differences, even after accounting for potentially lower rates in the e-visit group at six months than seen at three months.

### Aim 1 effectiveness trial data analytic plan

Main outcomes of the Aim 1 effectiveness trial include 1) evidence-based smoking cessation treatment utilization (medication, psychosocial cessation counseling), 2) reduction in cigarettes per day, and 3) biochemically verified 7-day PPA at six-month follow-up. Simple descriptive statistics will be calculated overall and within group for baseline demographic variables (e.g., sex, age, race, ethnicity, income, marital status, nicotine dependence, motivation to quit, other household members who smoke) and primary smoking-related outcomes. Baseline variables will be compared between treatment groups using Chi-square/Fisher’s exact tests (categorical variables) and t-tests or non-parametric equivalents (continuous variables), as appropriate, to identify potential covariates. Generalized linear mixed models (GLMMs) with logit links for binary outcomes will also be used to examine between group differences in baseline variables while accounting for provider and clinic clustering effects. As rates of the primary outcomes are expected to be low in the TAU group, we will utilize Fisher’s exact tests to compare rates between the e-visit and TAU groups at the one, three, and six-month follow-up assessments. To examine group differences adjusted for relevant covariates, GLMMs with logit links for binary outcomes will be used. These mixed methods models will account for any clustering effects within provider by including a random provider effect in the models. Clinic level effects will be examined in a similar manner.

#### Secondary group analyses

GLMMs including main effects of treatment and specific subgroup variables of interest (e.g., education, race, income, rurality, mental health comorbidities) along with an interaction term between treatment and the subgroup will be used to evaluate for which groups the e-visit is most beneficial. Each subgroup will be evaluated individually. All models will include a random provider effect to account for clustering. As this is an exploratory analysis, the focus will be on effect sizes rather than statistical significance.

#### Missing data and dropout

All enrolled participants will be included in analyses (intent-to-treat approach). We will examine dropout as function of treatment group to examine whether treatment is associated with differential study retention. A sensitivity analysis will be used to assess the potential effect of missing outcome data on parameter estimates. Parameters will be estimated using: 1) all available data, 2) missing outcome data imputed to baseline, and 3) methods of multiple imputations. Imputation of missing data in smoking cessation trials to the baseline condition is often used because it is a conservative approach [51], does not necessitate the missing and random assumption, and allows for correlation between missing status and smoking status [52].

### Aim 2 implementation evaluation

The secondary aim of this research is to provide an in-depth understanding of the acceptability, adoption, and sustainability outcomes of the e-visit intervention. A mixed-methods approach will assess implementation of the e-visit intervention throughout the effectiveness trial at the patient, provider, and organizational levels. Implementation factors are guided by the Consolidated Framework for Implementation Research (CFIR), which provides a comprehensive, pragmatic approach to understand implementation barriers, facilitators, and processes [53]. Further, the CFIR offers an organizational framework for synthesizing knowledge about an intervention across multiple settings. Specific implementation outcomes will be assessed according to Proctor’s Framework [27], which has been adapted for digital intervention evaluation [54]. These models suggest the evaluation of key implementation factors including acceptability, adoption, fidelity, cost, penetration, and sustainability. All self-report assessments will be administered to participants in the e-visit condition at the three-month research assessment, following completion of their e-visit (baseline and one-month). Provider questionnaires will be administered via REDCap to MUSC PCPs affiliated with clinics randomized to the e-visit condition who have at least one patient enrolled in the study. Provider questionnaires will be collected at six-months after the start of the study and again at the end of participant enrollment. After the implementation period, key informant interviews will be conducted with patients, PCPs, and healthcare leaders and qualitatively analyzed to enhance quantitative data.

#### Acceptability

We define acceptability as the degree to which the e-visit intervention is agreeable, palatable, and/or satisfactory to patients and providers. Intervention acceptability will be assessed via patient and provider self-report using the 4-item Acceptability of Intervention Measure [55]. Items are scored on a 5-point Likert scale, and the resulting score is the mean of responses.

#### Adoption

Adoption refers to the intention, decision, and/or initiation of the use of the e-visit intervention. At the patient level, we will capture the percent of: 1) e-visits opened, 2) e-visits completed and forwarded to the PCP, and 3) patients prescribed a medication who obtain their medication. At the provider level, we will assess the percent of: 1) e-visits opened by the patient’s PCP, 2) e-visits responded to by the patient’s PCP, and 3) e-visits that result in a medication prescription from the patient’s PCP. This approach will identify adoption gaps which will be further explored during key informant interviews.

#### Fidelity

We define fidelity as the extent to which the e-visit intervention is used as it is intended. Protocol adherence will be assessed at patient and provider levels. We will use an implementation tracking checklist to monitor completion of each step of the e-visit process for patients and providers. Research personnel will complete the checklist for each completed e-visit and we will evaluate the percentage of total steps completed. Across e-visits, we will assess which steps are most often skipped, which will be probed during key informant interviews and will guide refinements. For example, if across e-visits PCPs are e-prescribing medications but are not responding to patients electronically with treatment plans, this would suggest needed intervention modifications to augment and facilitate this process. During key informant interviews with PCPS, we will explore their perspectives on incomplete e-visit follow-ups as well as about their overall experience. Information gathered from these PCP interviews will be used to further refine the intervention.

#### Implementation costs

To examine cost-savings, a cost–benefit analysis will compare differences in all-cause and tobacco health-related healthcare expenditures prior to and following e-visit implementation relative to TAU. From this difference, we will subtract the cost of implementing e-visits and add anticipated revenues of $25 per e-visit. Cost data for all inpatient, outpatient, and emergency department care will be obtained from MUSC billings. As study participants may seek care outside of MUSC, all-payors’ claims data from South Carolina’s Revenue and Fiscal Affairs Office (RFA) will also be obtained. E-visit implementation cost will be provided by MUSC’s BMIC, who will provide ranges of e-visit development and distribution costs. Cessation medication costs will be based on actual billing data captured as part of the effectiveness trial but will account for national differences in costs using National Average Drug Acquisition Cost data. We will also vary anticipated revenues from $15.52 to $50.16 (the current Medicare e-visit reimbursement range) per patient in sensitivity analyses. The case for e-visit adoption and implementation will be the strongest if the e-visit reduces healthcare expenditures while at least providing non-inferior cessation outcomes [56].

For cost-effectiveness analyses, gold standard procedures [56] will be used to calculate the incremental cost effectiveness ratio (ICER), defined as the additional cost per additional desired outcome, operationalized as 7-day PPA at six-months. This study’s ICER is defined as: (cost of e-visit – cost of TAU)/ (e-visit 7-day PPA prevalence – TAU 7-day PPA prevalence), assuming the two groups (e-visit, TAU) have similar characteristics. If group differences are evident, a generalized linear model will be used to adjust outcomes and costs for between-groups differences. If the e-visit is more expensive with a less desirable result, it will be considered not cost effective. Otherwise, a probabilistic sensitivity analysis will test results robustness with differing ranges of costs, revenues, and treatment effectiveness [57]. Effectiveness ranges will be based on confidence intervals estimated in Aim 1 outcomes. Cost and revenue data, including potential ranges, will be captured in the manner described for the cost–benefit analysis. All costs and revenues will be converted to net present value at standard discount rates (3% and 5%). Based on the probabilistic sensitivity analysis, an acceptability curve will demonstrate the probability of the e-visit being cost-effective under different levels of willingness to pay.

#### Penetration

Penetration refers to the integration of a practice into a service setting. To assess the integration of the e-visit into these primary clinics, we will first determine the total number of unique PCPs employed by MUSC primary care clinics who reviewed a study e-visit and divide this number by the total number of PCPs employed by a clinic randomized to the e-visit condition. Patient-level penetration will be assessed during the sustainability evaluation period (12-month period beginning at enrollment completion [see next section]) and will be defined as the total number of unique patients who complete a smoking cessation e-visit divided by the total number of adult patients who are currently smoking cigarettes with MyChart access that have a primary care appointment.

#### Sustainability

Following recruitment completion of the effectiveness trial, a 12-month e-visit sustainability evaluation period will begin. During the final three months of trial recruitment, the study team (JD, VD, MP) will ensure that the e-visit (previously utilized for research), will be readied for clinical implementation across MUSC clinics. At the beginning of the sustainability evaluation period, the e-visit will become available for clinical utilization. During this year, providers will be able to invite their own patients to complete the e-visit. All training materials developed for providers in the context of the effectiveness trial will remain available during the sustainability evaluation period but will be modified to instruct providers on how to proactively invite their patients to complete the e-visit. Automated procedures to remind invited patients of the available e-visit will be clinically deployed. During the sustainability evaluation period, EHR analytics data will track adoption, fidelity, and penetration. E-visit costs will be billed consistent with standard institutional billing procedures (e.g., either to insurance or at a flat rate of $25 billed to the patient), and we will assess whether the difference in cost (free during effectiveness trial vs. cost during sustainability evaluation) impacts patient-level adoption by comparing adoption metrics during years of effectiveness trial vs. sustainability evaluation period and via key informant interviews.

#### Qualitative data collection and analysis

Key informant interviews will supplement quantitative data collection. Interviews will be conducted with patients (*n* = 30), PCPs (*n* = 20), and healthcare leaders (*n* = 5). Although quantitative data will identify implementation outcomes, qualitative data can evaluate and provide guidance on barriers and facilitators to implementation outcomes to guide future optimization. Diverse patients and PCPs in terms of demographics, time in practice, and cessation outcomes will be recruited for interviews. For patient interviews, we will specifically recruit patients who were invited to enroll in the trial, but opted not to (*n* = 10), patients who enrolled but did not complete either the baseline or one-month e-visit (*n* = 10), and patients who enrolled and completed both the baseline and one-month e-visits (*n* = 10). Similarly, we will recruit PCPs with high adoption of the e-visit (i.e., responded to > 80% of e-visits completed by their patients; *n* = 10), and low e-visit adoption (i.e., responded to < 20%; *n* = 10). PCPs and healthcare leaders will be recruited for these interviews via targeted e-mail and phone messages. Interviews (30–45 min) will be conducted by study team investigators in person or by telephone using a structured interview guide developed using CFIR constructs [53]. Interviews will focus on each implementation factor to enhance quantitative data within each domain. Interviews will be conducted until theme saturation is achieved [58] and will be audio-taped and transcribed for analysis. Methods to ensure trustworthiness of qualitative data collection and analysis (e.g., audit trails, prolonged engagement with data) will be used [59]. Qualitative data will be analyzed using NVivo software [60] and with a deductive/inductive template analysis approach [61]. Two coders will independently review and code data using an iterative, team-based process to refine the codebook with discrepancies resolved by the study team. After completing qualitative and quantitative data analysis independently, data from each source will be synthesized using graphical matrix configurations for data triangulation [62]. Qualitative themes will be supplemented by patterns identified in quantitative results. Findings will characterize needs, concerns, and impressions of key informants and will guide implementation strategies for disseminating the e-visit intervention widely.

## Discussion

This paper describes an ongoing Hybrid Type I effectiveness-implementation trial funded by the National Cancer Institute that aims to comprehensively assess effectiveness and implementation outcomes of a proactive e-visit intervention delivered via the EHR. Across 18 primary care practices, this trial will provide detailed information to inform future dissemination of this smoking cessation intervention in primary care settings broadly. This research builds upon our previous pilot work [26], with the overall goal to scale a proactive e-visit intervention beyond one academic medical center to dramatically improve cessation rates among adults who smoke cigarettes.

Even if study results demonstrate that e-visit effectiveness is no different from TAU, or if implementation outcomes suggest considerable refinements are needed, outcomes can inform considerations in the delivery of other proactive treatment approaches and remote clinical trial methodology. Specifically, implementation outcomes will detail how to refine and improve the provision of smoking cessation e-visits within the context of primary care from patient, provider, and organizational perspectives. Further, this trial will employ and assess the implementation of fully remote procedures for participation, including remote biochemical verification of smoking status [48]. Decentralized clinical trials that employ remote methodology, such as this trial, have potential to reduce traditional barriers (e.g., transportation issues, geographic distance, physical challenges) to participation and can increase accessibility of clinical research [63]. Evaluating remote biochemical verification within a large-scale effectiveness trial can improve the rigor of this methodology in future smoking cessation studies.

Several design decisions in this Hybrid Type I effectiveness-implementation trial were carefully considered. For one, participant-level randomization was opted against due to concerns regarding within-provider and within-clinic contamination of treatment effects. Therefore, participating clinics are randomized at trial outset. Second, to minimize the impact of changes over time on trial outcomes, all clinics serve as recruitment sites for the entire trial duration vs. enrolling one clinic at a time until each reaches its enrollment target. Third, all clinics within this trial are affiliated with one academic medical center (MUSC), which has a previously established e-visit program. Evaluating e-visit implementation within other care systems without e-visit programs is important to assess but beyond the scope of this Hybrid I trial. If implementation outcomes of this trial are promising, the next step is to purse implementation evaluation in more diverse settings via a Type II Hybrid trial.

## Conclusion

This trial will provide rich data at patient, provider, and organizational levels. The asynchronous proactive e-visit intervention evaluated in this study has great potential to reduce tobacco morbidity and mortality by improving uptake of evidence-based cessation treatment in primary care.

### Supplementary Information

**Additional file 1.**

## Data Availability

Not applicable.

## References

[1] U.S. Department of Health and Human Services (2020). Smoking cessation. A Report of the Surgeon General.

[2] U.S. Department of Health and Human Services. The Health Consequences of Smoking-50 Years of Progress: A Report of the Surgeon General. Atlanta (GA): Centers for Disease Control and Prevention; 2014. 24455788

[3] Cornelius ME, Loretan CG, Jamal A, Davis Lynn BC, Mayer M, Alcantara IC (2023). Tobacco product use among adults - United States, 2021. MMWR Morb Mortal Wkly Rep.

[4] Babb S, Malarcher A, Schauer G, Asman K, Jamal A (2017). Quitting smoking among adults - United States, 2000–2015. MMWR Morb Mortal Wkly Rep.

[5] Fiore MC, Jaen CR, Baker T (2009). Treating tobaco use and dependence: 2008 update.

[6] Jamal A, Dube SR, Malarcher AM, Shaw L, Engstrom MC (2012). Tobacco use screening and counseling during physician office visits among adults–National Ambulatory Medical Care Survey and National Health Interview Survey, United States, 2005–2009. MMWR Suppl.

[7] Juster HR, Ortega-Peluso CA, Brown EM, Hayes KA, Sneegas K, Gopez G (2019). A media campaign to increase health care provider assistance for patients who smoke cigarettes. Prev Chronic Dis.

[8] Schnoll RA, Rukstalis M, Wileyto EP, Shields AE (2006). Smoking cessation treatment by primary care physicians: an update and call for training. Am J Prev Med.

[9] Kruger J, O’Halloran A, Rosenthal A (2015). Assessment of compliance with U.S. Public Health Service clinical practice guideline for tobacco by primary care physicians. Harm Reduct J.

[10] Papadakis S, McDonald P, Mullen KA, Reid R, Skulsky K, Pipe A (2010). Strategies to increase the delivery of smoking cessation treatments in primary care settings: a systematic review and meta-analysis. Prev Med.

[11] Park ER, Gareen IF, Japuntich S, Lennes I, Hyland K, DeMello S (2015). Primary care provider-delivered smoking cessation interventions and smoking cessation among participants in the national lung screening trial. JAMA Intern Med.

[12] Boyle R, Solberg L, Fiore M (2014). Use of electronic health records to support smoking cessation. Cochrane Database Syst Rev.

[13] Bailey SR, Heintzman JD, Marino M, Jacob RL, Puro JE, DeVoe JE (2017). Smoking-cessation assistance: before and after stage 1 meaningful use implementation. Am J Prev Med.

[14] McCarthy DE, Adsit RT, Zehner ME, Mahr TA, Skora AD, Kim N (2020). Closed-loop electronic referral to SmokefreeTXT for smoking cessation support: a demonstration project in outpatient care. Transl Behav Med.

[15] Richter KP, Shireman TI, Ellerbeck EF, Cupertino AP, Catley D, Cox LS (2015). Comparative and cost effectiveness of telemedicine versus telephone counseling for smoking cessation. J Med Internet Res.

[16] Haas JS, Linder JA, Park ER, Gonzalez I, Rigotti NA, Klinger EV (2015). Proactive tobacco cessation outreach to smokers of low socioeconomic status: a randomized clinical trial. JAMA Intern Med.

[17] Nelms E, Wang L, Pennell M, Wewers ME, Seiber E, Adolph MD (2014). Trust in physicians among rural Medicaid-enrolled smokers. J Rural Health.

[18] Lee YY, Lin JL (2009). The effects of trust in physician on self-efficacy, adherence and diabetes outcomes. Soc Sci Med.

[19] Erdmann M, Edwards B, Adewumi MT (2022). Effect of electronic portal messaging with embedded asynchronous care on physician-assisted smoking cessation attempts: a randomized clinical trial. JAMA Netw Open.

[20] Habermann EB (2019). Advancing the science of e-visits in primary care. Mayo Clin Proc.

[21] Zhong X, Hoonakker P, Bain PA, Musa AJ, Li J (2018). The impact of e-visits on patient access to primary care. Health Care Manag Sci.

[22] Green LV, Savin S, Lu Y (2013). Primary care physician shortages could be eliminated through use of teams, nonphysicians, and electronic communication. Health Aff.

[23] Murray E, Burns J, See TS, Lai R, Nazareth I (2005). Interactive health communication applications for people with chronic disease. Cochrane Database Syst Rev.

[24] de Jong CC, Ros WJ, Schrijvers G (2014). The effects on health behavior and health outcomes of Internet-based asynchronous communication between health providers and patients with a chronic condition: a systematic review. J Med Internet Res.

[25] Dixon RF, Rao L (2014). Asynchronous virtual visits for the follow-up of chronic conditions. Telemed J E Health.

[26] Dahne J, Player M, Carpenter MJ, Ford DW, Diaz VA (2021). Evaluation of a proactive smoking cessation electronic visit to extend the reach of evidence-based cessation treatment via primary care. Telemed J E Health.

[27] Proctor EK, Landsverk J, Aarons G, Chambers D, Glisson C, Mittman B (2009). Implementation research in mental health services: an emerging science with conceptual, methodological, and training challenges. Adm Policy Ment Health.

[28] Proctor E, Silmere H, Raghavan R, Hovmand P, Aarons G, Bunger A (2011). Outcomes for implementation research: conceptual distinctions, measurement challenges, and research agenda. Adm Policy Ment Health.

[29] Harris PA, Taylor R, Minor BL, Elliott V, Fernandez M, O'Neal L (2019). The REDCap consortium: Building an international community of software platform partners. J Biomed Inform.

[30] Harris PA, Taylor R, Thielke R, Payne J, Gonzalez N, Conde JG (2009). Research electronic data capture (REDCap)–a metadata-driven methodology and workflow process for providing translational research informatics support. J Biomed Inform.

[31] Patridge EF, Bardyn TP (2018). Research electronic data capture. J Med Libr Assoc.

[32] Health Resources & Services Administration. What Is Shortage Designation? 2023. Available from: https://bhw.hrsa.gov/workforce-shortage-areas/shortage-designation#hpsas.

[33] Chen C, Turner SP, Sholle ET, Brown SW, Blau VLI, Brouwer JP (2019). Evaluation of a REDCap-based workflow for supporting federal guidance for electronic informed consent. AMIA Jt Summits Transl Sci Proc.

[34] Cropsey KL, Bean MC, Haynes L, Carpenter MJ, Richey LE (2020). Delivery and implementation of an algorithm for smoking cessation treatment for people living with HIV and AIDS. AIDS Care.

[35] Cropsey KL, Jardin BF, Burkholder GA, Clark CB, Raper JL, Saag MS (2015). An algorithm approach to determining smoking cessation treatment for persons living with HIV/AIDS: results of a pilot trial. J Acquir Immune Defic Syndr.

[36] Valera P, McClernon FJ, Burkholder G, Mugavero MJ, Willig J, O'Cleirigh C (2017). A pilot trial examining African American and white responses to algorithm-guided smoking cessation medication selection in persons living with HIV. AIDS Behav.

[37] National Cancer Institute. Clearing the air: Quit smoking today 2003. Available from: https://www.cancer.gov/publications/patient-education/clearing-the-air.

[38] Roque NA, Boot WR (2018). A new tool for assessing mobile device proficiency in older adults: the mobile device proficiency questionnaire. J Appl Gerontol.

[39] Norman CD, Skinner HA (2006). eHEALS: the eHealth literacy scale. J Med Internet Res.

[40] Boot WR, Charness N, Czaja SJ, Sharit J, Rogers WA, Fisk AD (2015). Computer proficiency questionnaire: assessing low and high computer proficient seniors. Gerontologist.

[41] Kroenke K, Strine TW, Spitzer RL, Williams JB, Berry JT, Mokdad AH (2009). The PHQ-8 as a measure of current depression in the general population. J Affect Disord.

[42] Sobell L, Sobell M (1996). Timeline followback user’s guide: a calendar method for assessing alcohol and drug use Toronto.

[43] Brown RABE, Sales SD, Whiteley JA, Evans DM, Miller IW (1998). Reliability and validity of a smoking timeline follow-back interview. Psychol Addict Behav.

[44] Heatherton TF, Kozlowski LT, Frecker RC, Fagerstrom KO (1991). The fagerstrom test for nicotine dependence: a revision of the fagerstrom tolerance questionnaire. Br J Addict.

[45] Biener L, Abrams DB (1991). The Contemplation Ladder: validation of a measure of readiness to consider smoking cessation. Health Psychol.

[46] Perkins KA, Karelitz JL, Jao NC (2013). Optimal carbon monoxide criteria to confirm 24-hr smoking abstinence. Nicotine Tob Res.

[47] Vijayaraghavan M, Yuan P, Gregorich S, Lum P, Appelle N, Napoles AM (2017). Disparities in receipt of 5As for smoking cessation in diverse primary care and HIV clinics. Prev Med Rep.

[48] Dahne J, Tomko RL, McClure EA, Obeid JS, Carpenter MJ (2020). Remote methods for conducting tobacco-focused clinical trials. Nicotine Tob Res.

[49] Carpenter MJ, Wahlquist AE, Dahne J, Gray KM, Garrett-Mayer E, Cummings KM (2020). Nicotine replacement therapy sampling for smoking cessation within primary care: results from a pragmatic cluster randomized clinical trial. Addiction.

[50] Dahne J, Lejuez CW, Diaz VA, Player MS, Kustanowitz J, Felton JW (2019). Pilot randomized trial of a self-help behavioral activation mobile app for utilization in primary care. Behav Ther.

[51] Hall SM, Delucchi KL, Velicer WF, Kahler CW, Ranger-Moore J, Hedeker D (2001). Statistical analysis of randomized trials in tobacco treatment: longitudinal designs with dichotomous outcome. Nicotine Tob Res.

[52] Barnes SA, Larsen MD, Schroeder D, Hanson A, Decker PA (2010). Missing data assumptions and methods in a smoking cessation study. Addiction.

[53] Damschroder LJ, Aron DC, Keith RE, Kirsh SR, Alexander JA, Lowery JC (2009). Fostering implementation of health services research findings into practice: a consolidated framework for advancing implementation science. Implement Sci.

[54] Hermes ED, Lyon AR, Schueller SM, Glass JE (2019). Measuring the implementation of behavioral intervention technologies: recharacterization of established outcomes. J Med Internet Res.

[55] Weiner BJ, Lewis CC, Stanick C, Powell BJ, Dorsey CN, Clary AS (2017). Psychometric assessment of three newly developed implementation outcome measures. Implement Sci.

[56] Weinstein MC, Siegel JE, Gold MR, Kamlet MS, Russell LB (1996). Recommendations of the Panel on Cost-effectiveness in Health and Medicine. JAMA.

[57] Drummond MF SM, Claxton K, Stoddart GL, Torrance GW. Methods for the economic evaluation of health care programmes. Oxford university press; 2015.

[58] Saunders B, Sim J, Kingstone T, Baker S, Waterfield J, Bartlam B (2018). Saturation in qualitative research: exploring its conceptualization and operationalization. Qual Quant.

[59] Korstjens I, Moser A (2018). Series: practical guidance to qualitative research. Part 4: trustworthiness and publishing. Eur J Gen Pract.

[60] QSR International Pty Ltd. NVivo (released in March 2020). 2020.

[61] Brooks J, McCluskey S, Turley E, King N (2015). The utility of template analysis in qualitative psychology research. Qual Res Psychol.

[62] Guetterman TC, Fetters MD, Creswell JW (2015). Integrating quantitative and qualitative results in health science mixed methods research through joint displays. Ann Fam Med.

[63] Dahne J, Hawk LW (2023). Health equity and decentralized trials. JAMA.

